# On Starting a Medical Library

**Published:** 1913-02-15

**Authors:** 


### ON STARTING A MEDICAL LIBRARY.

When the student has received his diploma and goes to his first locum engagement he finds, perhaps for the first time, that he has a need of certain books. It is not because he does not know his work, but because, perhaps, he knows for the moment just a little too much of certain aspects of that work. These are aspects which were held up to him in his student days as first aspects; they are mainly considerations of treatment, of diagnosis, and of pathology. But very soon he finds that there are other and equally important considerations. His relations with fellow-practitioners and with patients, his ignorance of the whole gamut of medical etiquette, his treatment of trivial cases which persist in bothering him, although they never caused him much worry in the out-patient department, where he had the assistance and experience of seniors to fall back upon—all these combine to give him mental pain. He finds, very probably, that his principal has a small and strikingly obsolete collection of technical books, in which are laid down lines of treatment which would now be regarded as directly dangerous. What he learns in his locum time is of value to him, for if he is wise he will start at once in collecting the nucleus of his own library.

Of his student books probably the majority will be cast into the hands of the nearest second-hand dealer. A few useful ones he will retain, both for the sake of old associations and for their intrinsic merits. Thus his dissecting-room manual, if it is a more or less new copy, will remain; his Gray or Morris he cannot part with, for if he is wise he will devote some attention to their pages in leisure moments; his Taylor or Osler, useful enough for examination purposes and for handy reference, he will also do well to keep, but he will be wise in reinforcing them by the purchase of some good compendium, such as Allbutt's system or Osler and Macrae. One of these sets, with a good modern text-book on treatment—Burney Yeo or the new edition of Latham and English for preference—will give him much solace in time of trouble. Perhaps also he should append to his stock one or two manuals dealing with special diseases. A good text-book on the diseases of children—Eustace Smith, Ashby and Wright, and Goodhart and Still are all excellent authorities, but best of all, perhaps, Pfaundler and Schlossmann, if the collector can afford the additional outlay—will be helpful. Ditto one on fevers, another, perhaps, on heart disease, and a third on consumption. But if he is fortunate to possess one of the all-embracing medical "Systems" he can afford to dispense with these extra aids. Let him before all beware of out-of-date second-hand books.

In surgery he will find that his student text-book serves as well as any other. Perhaps his best course will be to strengthen this line by acquiring a good manual of surgical treatment and after-treatment; undoubtedly the best work for him here is the new edition of Cheyne and Burghard. The new surgical "System" edited by Choyce is an admirable book to possess; if the practitioner knows German, probably the best addition to his library that he can make is the set—or some volumes out of it—of the fine monographs published under the general title of "Deutsche Chirurgie"; these are undoubtedly the best works on surgery extant, and they give such excellent bibliographies that they are a library in themselves. A few special books are necessary here as well—one on diseases of the eye, one or two on gynæcology and obstetrics, and one on diseases of the skin.

So far for what are purely the professional aspects of the library. But it must not be thought that when he has all these text-books and assistance that he will possess a complete medical library. "The Medical Register" he will, of course, possess; it comes to him as the direct result of his paying the registration fee and the usual extra, which in his joy at passing he is hardly disposed to cavil at. "The Medical Directory" is an even more useful possession, and he would be well advised to get a copy, either regularly every year or every two or three years. It keeps him in touch with his old fellow-students, and it shows him what is doing in the medical world in which he moves. Similarly he would do well to subscribe to a good medical paper. Here, again, if he reads a foreign language, he might with advantage take in a foreign medical journal; such papers as the Berlin *Klinik* or the Munich *Wochenschrift* are mines of up-to-date information, the perusal of which will keep him abreast of the times. In England the only medical paper that can compare with either of these is the *Lancet*, and we take it that no medical man who can afford to pay sixpence a week for a paper fails to read that journal.

In one or two other directions the library may be usefully amplified. It should contain, for example, a copy of some good book on medical ethics; we know of none better for the junior practitioner than Professor Saundby's little volume. A few books on current questions of the day that involve medical interests should be added. Copies of some particular acts, for example, should be on the shelves; a good manual of hygiene and one of the Poor-Law manuals may also be added. Now that the Insurance Act has come to stay it is needless to say that some manual dealing with this recent legislation should be included in the collection. Finally, there are some works which, although they do not primarily appeal to the practitioner himself, are yet supremely useful, inasmuch as they are popular expositions which are valuable to teach patients certain essentials—a small book on the treatment and training of children, one on diet, another on sanatoria—the choice, indeed, in this direction is limited only by the personal prejudices and tastes of the practitioner.

The acquisition of such a small library as we have rapidly sketched in this article does not entail a considerable outlay. By discriminate selection a fully representative collection of new books of the types we have mentioned may be purchased for less than £25. Of course, if elaborate systems and foreign works are added, this figure will be increased. But there is no necessity to buy the whole collection at once. Let the practitioner study his own needs, and purchase from time to time such books as he requires.

We have made no mention in this summary of The Hospital; yet the practitioner will find it to his advantage to subscribe to this paper, especially if he is in a position which brings him into relation with institutional interests. Not primarily a medical journal in the sense that the journals we have mentioned are, The Hospital nevertheless appeals to the practitioner on two main grounds—one is in that it endeavours to give from time to time a judicious and carefully compiled synopsis of new methods and of the general progress of medicine; another because it strives to bring into relation with one another the different interests of all sections of workers in the medical and institutional field. In both respects the practitioner will find it a highly helpful paper, and its subscription is so low that there should be no excuse for omitting to subscribe to it.

